# A Technical Report on the Performance of a New Large Bore Cerebral Aspiration Catheter, the First Results

**DOI:** 10.1007/s00270-019-02280-z

**Published:** 2019-07-02

**Authors:** Faysal Benali, Robert-Jan B. Goldhoorn, Bart A. J. M. Wagemans, Christiaan van der Leij, Rutger J. B. Brans, Sanne W. de Boer, Michiel W. de Haan, Wim H. van Zwam

**Affiliations:** 1 Department of Radiology, Maastricht University Medical Center + (MUMC +), P. Debyelaan 25, 6229 HX Maastricht, The Netherlands; 2 Department of Neurology, Maastricht University Medical Center + (MUMC +), Maastricht, The Netherlands

**Keywords:** Neuro-intervention, Stroke, Aspiration, Interventional radiology

## Abstract

**Aims and Background:**

We describe the initial results of the Syphontrack Super Distal Access (SDA) catheter (InNeuroco Inc., Sunrise, Fl, USA) used for endovascular treatment of patients with acute ischemic stroke of the anterior circulation.

**Methods:**

A retrospective review of prospectively collected data from June 2017 to May 2018 in Maastricht University Medical Center plus (MUMC +) with direct distal aspiration or a combination of distal aspiration with stent retriever thrombectomy was performed. Primary outcome measurements were accessibility and reperfusion grade (eTICI). Secondary outcome measurements were early neurologic recovery (a decrease of four or more points on the NIHSS), symptomatic intracranial hemorrhage (sICH) within 24 h and mRS score at 3 months.

**Results:**

The first 50 patients in whom the SDA catheter was used are included. Direct distal aspiration was performed in 33/50 (66%). In 29/33 (88%), distal position in contact with the clot was achieved of which 15 (52%) were successful (eTICI 2b or higher) after first attempt. Total successful reperfusion rate was 23/50 (46%) after first pass. Final successful reperfusion, after multiple attempts, was reached in 48/50 (96%). Early neurologic recovery was seen in 21/50 (42%), and functional independence (mRS score of 0–2) at 3 months was achieved in 17/50 (35%). sICH occurred in 4/50 (8%) within 24 h post-procedural.

**Conclusion:**

In our clinical practice, endovascular treatment of ischemic stroke with the SDA catheter had similar technical and clinical results as reported in the literature.

## Introduction

The large bore Syphontrack Super Distal Access (SDA) catheter (InNeuroCo, Sunrise, FL, USA) is a modification of the Syphontrack intermediate catheter of which the use as a supportive device has been recently published [1]. The SDA catheter is designed to perform direct aspiration of the clot and to provide stable support in a triaxial thrombectomy approach. It has a rigid proximal part and a flexible distal part with a lubricous coating, allowing for easy navigability and stable support. Working length is 135 cm (53.15 inch), inner diameter is 1.57 mm (0.06 inch) and proximal/distal outer diameters are 2.11 mm (0.08 inch) and 1.93 mm (0.075 inch), respectively. A major advantage is that the SDA catheter can fit into an 8F Flowgate catheter [Stryker neurovascular, Fremont, Ca, USA], where other 6F catheters need a 9F balloon guide catheter. We report performance of the SDA in a direct distal aspiration technique (where it is placed in contact with the thrombus), as well as in a combined approach (where it can be located more proximal). Since the focus of this study is on the technical performance of this new catheter, the clinical aspects are not outlined in detail.

## Methods

This study is a retrospective analysis of prospectively collected data from MUMC + over a period of 12 months (June 2017–May 2018).

### Inclusion Criteria

All cases eligible for endovascular thrombectomy (according to current guidelines) and performed with SDA catheter were included.

### Endovascular Procedure

In our hospital, EVT procedures are primarily performed under local anesthesia and if necessary in combination with conscious or general sedation (e.g., in case of excessive patient movement). Endovascular procedures are performed by accredited and experienced (neuro)-interventional radiologists. After local anesthesia, the common femoral artery is usually accessed under ultrasound guidance and an 8F or 9F sheath is inserted. An 8F or 9F balloon guide catheter (Flowgate 8 F or Merci 9 F, [Stryker neurovascular, Fremont, Ca, USA]) is positioned in the internal carotid artery after which the occlusion site is confirmed by angiography. In case a femoral access is not possible (for example due to severe tortuosity and/or occlusion), access is acquired through direct carotid puncture after which a 6F sheath is inserted. Thereafter, the SDA, together with a microcatheter (Orion 21, Medtronic [Irvine, Ca, USA] or Trevo 18, Stryker) and microwire (Transend 0.014, Stryker), is placed in the occluded vessel. Subsequently, the SDA is placed just proximal to the thrombus and manual aspiration is performed using two syringes of 60 cc. If the SDA catheter does not reach the clot, the procedure is continued with a stent retriever (Solitaire [Medtronic, Fridley, Min, USA], Revive or Embotrap [Cerenovus, Irvine, Cal, USA]), placed in and beyond the clot. A combined proximal aspiration and distal retraction is then performed. If it is the interventionist’s preference to start with the combined approach, the same steps are followed.

### Outcome Measurements

Primary outcome measurements were reperfusion grade and accessibility. The reperfusion grade was reported according to the “extended thrombolysis in cerebral infarction” (eTICI) scale [2, 3]. Successful reperfusion was defined as eTICI 2b or higher. Secondary outcome measurements were: time from groin puncture to reperfusion, occurrence of periprocedural ipsilateral emboli, early neurologic recovery (decrease of 4 or more points on the National Institutes of Health Stroke Scale (NIHSS) 24–48 h post-intervention), symptomatic intracranial hemorrhage (sICH) within 24 h according to Heidelberg criteria [4], functional outcome (modified Rankin scale (mRS) score) and mortality reported within 90-day follow-up.

### Subgroup Analysis

Subgroup analysis was performed on patients treated with direct distal aspiration and patients with a combined approach.

### Statistics

Descriptive statistics were used for analyzing baseline characteristics and outcomes. For subgroup analysis, chi-square testing or fisher’s exact was used. For continuous variables, the Mann–Whitney *U* test was used. All analyses were done with SPSS package version 25.

## Results

### Baseline Characteristics (Table 1)

Fifty consecutive patients were included. Mean age was 72. The occlusion was most frequently located in the ACM-M1 segment (*n* = 22, 44%). In 33/50 (66%) patients, direct distal aspiration was intended, the other 17 were started as combined approach.

**Table 1:** Baseline variables (*N* = 50)

Age, mean (SD)	72 (11)
Male sex, *n* (%)	33 (66)
Pre-stroke mRS score, median (IQR)	0 (0–2)
NIHSS score, median (IQR)	15 (11–17)
Location of occlusion, *n* (%)	
M1	22 (44)
M2	17 (34)
ICA	4 (8)
ICA-terminus	5 (10)
Tandem occlusions	2 (4)
Approach, *n* (%)	
Direct distal aspiration	33 (66)
Combined approach	17 (34)
Total time of procedure, mean minutes (SD)	54 (39)
Anesthetic management during procedure, *n* (%)	
Local anesthesia	40 (80)
General anesthesia	6 (12)
Conscious sedation	4 (8)
Access point, *n* (%)	
Femoral artery	47 (94)
Carotid artery	3 (6)

### Primary Outcome Measurements (Table 2)

In four out of 33 (12%) direct aspiration cases, the SDA did not reach the clot. This was due to tortuosity of the carotid arteries (2 cases), distress of the patient (1 case) and unknown reason (1 case). Final eTICI 2b or more was achieved in 48 out of 50 cases (96%). In 23 out of 50 (46%), this was achieved at first attempt. 52% (15/29) of direct aspiration at first attempt and 38% (8/21) of the combined technique (Table 3). After failed first attempt, number of consecutive aspiration attempts before switching to stentretriever were not documented. However, according to the radiology reports this ranged from 1 to “multiple.”

**Table 2:** Outcome (*N* = 50)

eTICI 2b–3 (after one attempt), *n* (%)	
Yes	23 (46)
No	27 (54)
eTICI (final), *n* (%)	
1	2 (4)
2b	7 (14)
2c	3 (6)
3	38 (76)
Occlusion reached when intended with direct distal aspiration, *n* (%)	
Yes	29 (88)
No	4 (12)
Time from groin puncture to successful direct distal aspiration, mean minutes (SD)	54 (39)
Ipsilateral emboli, *3* (%)	2 (4)
Successfully treated during procedure, *n* (%)	1 (50)
Other complications, *n* (%)	4 (8)
Early neurologic recovery, *n* (%)	
Improvement ≥ 4 points (NIHSS)	21 (42)
sICH within 24 hours, *n* (%)	4 (8)
Death at 90-day follow-up, *n* (%)	12 (24)
mRS score at 90-day follow-up*, *n* (%)	
0–1	12 (24)
0–2	17 (35)
> 2	32 (65)

*N = 49. 1 patient lost to follow-up

**Table 3:** Subgroup analysis: direct distal aspiration versus combined approach (*N* = 50)

	Direct distal aspiration (*N* = 29)	Combination (*N* = 21)	*P*-value
eTICI 2b - 3 (after one attempt), *n* (%)			
Yes	15 (52)	8 (38)	0.474
No	14 (48)	13 (62)	
eTICI 2b–3 (final), *n* (%)			
Yes	28 (97)	20 (95)	0.669
No	1 (3)	1 (5)	
sICH within 24 hours, *n* (%)	2 (7)	2 (9.5)	0.543
Death at 90-day follow-up, *n* (%)	6 (21)	6 (28.6)	0.585
Early neurologic recovery, *n* (%)			
Improvement ≥ 4 points (NIHSS)	11 (38)	10 (47.6)	0.233
mRS 90-day follow-up, *n* (%)	*		
0–1	7 (25)	5 (24)	0.364
0–2	12 (43)	5 (24)	
> 2	16 (57)	16 (76)	
Time from groin puncture to success, mean minutes (SD)	48 (40)	60 (36)	0.148
Ipsilateral emboli, *n* (%)	2 (6.9)	0 (0)	0.219
Other complications, *n* (%)	3 (10)	1 (4.8)	0.495

*N = 28.1 patient lost to follow-up

### Secondary Outcome Measurements and Safety (Table 2)

Periprocedural ipsilateral emboli in distal M3 segment were seen in 2/50 (4%) procedures, of which 1 was accessible for thrombectomy and successfully treated (Table 2). Other periprocedural complications documented (4/50) were mainly dissections of intracranial artery as well as extracranial carotid artery (*n* = 3) and luxation of the sheath (*n* = 1). These complications could be (indirectly) contributed to the catheter. One of these complications eventually led to abortion of the procedure. Final mRS 0–2 was reached in 17/49 (35%) patients.

### Subgroup Analysis (Table 3)

No significant differences between direct distal aspiration and the combined approach as first attempt were found.

## Discussion

Distal access catheters were initially designed as supportive catheters for various neuro-interventional procedures. Later, these supportive catheters appeared successful as an aspiration device for stroke treatment which led to the development of more dedicated aspiration catheters. The first devices developed, and mostly used in aspiration trials, are the Penumbra catheters (Penumbra, Almeda, CA, USA) [5]. The safety and effectiveness of these catheters were published in 2008–2009 [6]. In our hospital, the SOFIA (Microvention, Aliso Viejo, CA, USA) [7, 8] and the SDA catheter are currently the two most used devices. This is the first report on outcomes achieved with the SDA catheter as aspiration catheter in endovascular stroke treatment. Regarding the most important features of such catheter: navigability, efficiency, safety and clinical outcome, we found comparable results with the devices used in the major published trials such as ASTER and COMPASS [9, 10].

As mentioned in the analysis of Andersson et al. [11], risk of distal emboli is of particular importance when performing direct distal aspiration. Especially when no additional balloon guide catheter with flow arrest is performed. In our cases, this balloon guide flow arrest was not consequently done. However, exact data on balloon guide use in our cases are not documented. Nonetheless, the overall embolization rate is lower than in published data [7, 12–14].

## Limitations

We report data from a single center. Although the study was designed in retrospect, our data were collected prospectively. Reperfusion grade was scored by the interventionist at the end of the procedure and, consequently, could have been subject to bias.

## Conclusion

In our clinical practice, endovascular treatment of ischemic stroke with the SDA catheter showed good technical results. Clinical outcome of treated patients and safety were comparable to reports with use of other aspiration catheters.
